# Extra-Anogenital Giant Cutaneous Squamous Cell Carcinomas

**DOI:** 10.3390/life14030421

**Published:** 2024-03-21

**Authors:** Mateusz K. Mateuszczyk, Iwona Chlebicka, Magdalena Łyko, Joanna Maj, Jacek C. Szepietowski

**Affiliations:** Department of Dermatology, Venereology and Allergology, Wroclaw Medical University, 50-368 Wroclaw, Poland; mateuszczyk25@gmail.com (M.K.M.); iwonak4wsk@interia.pl (I.C.); magdalena.lyko@student.umw.edu.pl (M.Ł.); joanna.maj@umw.edu.pl (J.M.)

**Keywords:** giant cutaneous squamous cell carcinoma, cutaneous squamous cell carcinoma, squamous cell carcinoma

## Abstract

Extra-anogenital giant cSCCs are rare but have worse outcomes compared to smaller tumors. Prompted by limited data, the authors conducted a retrospective study to gather more information about giant cSCCs to optimize clinical care. We identified seven cases of giant cSCCs from a review of cSCC cases treated in the Unit of Dermatosurgery between 2016 and 2022. Most patients were male (85.71%) with a mean age of 80.29 ± 12.22 years. UV radiation was the most common risk factor (five cases) followed by smoking (three cases) and hidradenitis suppurativa (one case). Most giant cases were located in the head area (71.4%) and the diameter of lesions ranged from 6 to 18 cm. All patients corresponded to tumor stage T3, and 42.86% of patients had lymph node metastases. Surgical excision was the treatment of choice in most cases (85.71%), while a combination of cemiplimab and RP1 was used in some cases due to the ineffectiveness of treatment or contraindications to other therapies. The authors emphasize the importance of early detection and prevention of modifiable risk factors, such as UV radiation, and a multidisciplinary approach to treatment. Other therapies, including immunotherapy, may become increasingly important.

## 1. Introduction

Cutaneous squamous cell carcinoma (cSCC) is the second most common skin cancer arising from epidermal keratinocytes [1]. The number of cSCC cases has increased over recent years, making it a health issue of global importance [2]. cSCCs can become a giant tumor in case of delayed treatment due to not being noticed or being neglected by the patient [3]. By definition, a giant is referred to as a tumor whose maximum clinical diameter is at least 5 cm [4]. Extra-anogenital giant cSCCs are rarely reported in the literature. Considering the rarity and absence of prospective randomized trials, consistent recommendations for medical management do not exist [3]. Prompted by the limited data, we conducted this study to gather more information about giant cSCCs to optimize clinical care.

## 2. Materials and Methods

This study was based on a retrospective review of SCC cases treated at the Unit of Dermatosurgery of the Department of Dermatology, Venereology and Allergology of Wroclaw Medical University in Poland between January 2016 and June 2022. In the medical records of the hospital electronic database, we identified 96 cSCC cases using the International Classification of Diseases, 10th revision (ICD-10) code C44. Patient charts were thoroughly evaluated. Subjects over 18 years of age, with a diagnosis of SCC confirmed in histology, lesions located in the extra-anogenital area, and a diameter of the lesion of at least 5 cm, were included in our study. Localization in the anogenital region, smaller diameter, and lack of histology results were among the exclusion criteria. Patients fulfilling the criteria were enrolled for further analysis. Seven cases of extra-anogenital giant cSCC were identified during this period. In all cases, histology confirmed the verrucous subtype of SCC. We used standardized data collection. For each patient, data analyses included sex, age at the time of treatment, tumor location and size, identified risk factors, and time to diagnosis. Nodal metastasis was documented with diagnostic imaging and confirmed with lymph node dissection. Moreover, we carefully analyzed the treatment regimen and outcome in all subjects. For classification, we used the American Joint Committee on Cancer (AJCC)/International Union against Cancer (UICC) tumor–node–metastasis (TNM) staging system [1]. Due to the size of the study population, the statistical analysis was limited to descriptive statistical methods.

## 3. Results

### 3.1. Patients

**Figure 1:**
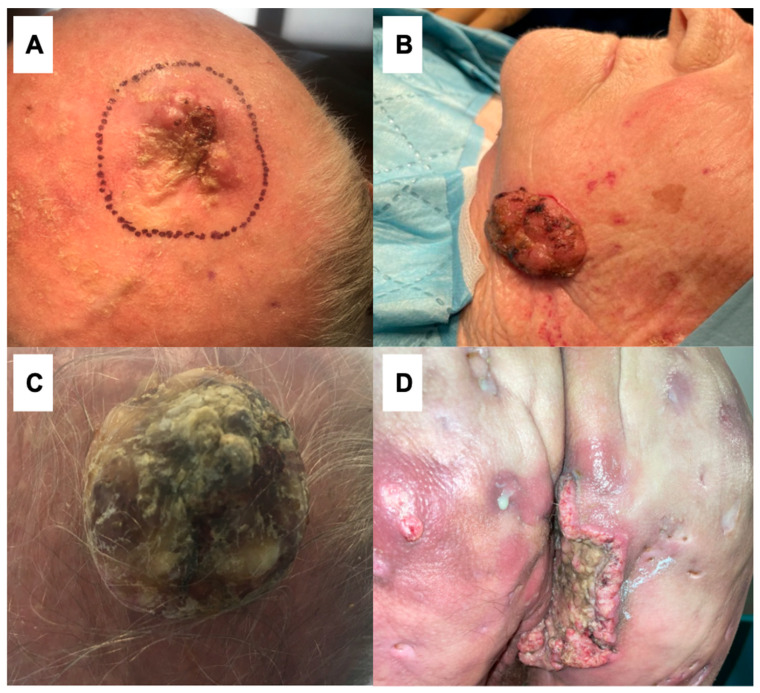
Clinical presentations of giant cutaneous squamous cell carcinomas: (**A**) 81 mm cSSC in an 89-year-old male located on the scalp; (**B**) 62 mm cSCC in an 83-year-old female located on the left cheek; (**C**) 115 mm cSCC in an 84-year-old male on the scalp; (**D**) 103 mm cSCC in a 59-year-old male with coexisting hidradenitis suppurativa located on the right buttock.

**Figure 2:**
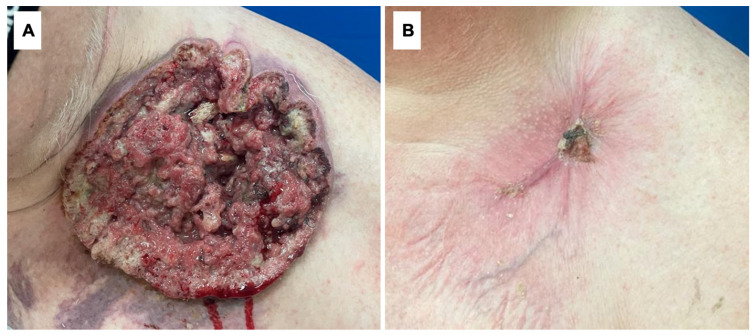
(**A**) A giant cutaneous squamous cell carcinoma located in the left supraclavicular region in a 68-year-old male. (**B**) The same tumor after 4 months of treatment with cemiplimab + RP1.

Among the 96 cSCCs treated at the Unit of Dermatosurgery, 7 (7.29%) cases of giant cSCCs were reported. In our study group, six (85.71%) out of seven patients were male. The age range was from 59 to 93 years with a mean age of 80.29 ± 12.22 years (mean ± standard deviation). The SCCs were localized on the scalp (three), forehead (one), cheek (one), supraclavicular area (one), and buttock (one). The mean time to diagnosis was 34.57 ± 17.19 months (from 12 to 36 months). Among the risk factors, UV radiation (five), smoking (three), and hidradenitis suppurativa (HS) (one) were reported. The delay in diagnosis was associated with patients’ neglect in all cases. Moreover, five patients were afraid of being diagnosed. One patient was diagnosed with hidradenitis suppurativa and clinical symptoms at the beginning mimicking an HS lesion, which contributed to the delay in diagnosis. The patients’ characteristics are summarized in Table 1, the clinical manifestations are given on Figure 1 and Figure 2.

### 3.2. Clinical Findings and Disease Stage 

The diameter of skin lesions ranged from 60 to 180 mm, and the mean size was 97.14 ± 39.04 mm. The size of the tumor ranged from 114.92 to 562.94 cm^2^ with a mean size of 273.81 ± 154.85 cm^2^ (Table 1). In all patients, the results showed a verrucous subtype of SCC. At presentation, all patients corresponded to tumor stage T3. Lymph node metastases were observed in three (42.86%) patients and neither of our patients was identified with distant metastasis (Table 2). Figure 1 illustrates the clinical presentations of the tumors.

### 3.3. Treatment and Outcome

In six (85.71%) out of seven patients, surgical excision was the treatment of choice. Of them, in four patients (57.14%) primary radical surgical excision was performed and R0 resection was achieved in all of them. Skin graft transplants were used for defect closure in three patients. In one remaining patient, with the smallest tumor, an excision with primary closure was performed. Despite the surgical treatment, in two patients we observed the recurrence of SCC. In one of them, radiotherapy was introduced with a good clinical response. However, after another recurrence, he was qualified to receive cemiplimab administered intravenously every 3 weeks in combination with RP1 administered as an intratumoral injection every 3 weeks for a year. The second patient, due to inoperable tumor size, was also qualified for immunotherapy. As a result of the treatment, the tumor significantly decreased in size. Figure 2 presents the treatment outcome in this patient after 4 months of therapy with cemiplimab and RP1. Moreover, one patient with coexisting HS was treated with cemiplimab and RP1 due to contraindications to other therapeutic approaches. In total, three patients were qualified for this alternative treatment. In two patients with nodal metastasis, regional lymph node dissection was performed (Figure 1, Table 2).

Four patients (57.14%) are alive without evidence of disease, two are alive with disease (28.57%) and currently receiving treatment, and one death of disease (14.29%) was reported. The treatment regimens and outcomes are summarized in Table 2. 

## 4. Discussion

Cutaneous squamous cell carcinoma (cSCC) is the second most common skin cancer arising from epidermal keratinocytes and accounts for approximately 20 percent of non-melanoma skin cancers (NMSCs) [1,5]. The number of cSCC cases has increased over the past years, making it a health issue of global importance [2]. The estimated lifetime risk of being diagnosed with cSCC is 4–9% for females and 9–14% for males [6]. However, the precise incidence of cSCCs, including giant cSCCs, remains unknown, partially due to the lack of cancer registries that collect data on cSCCs in most countries and frequently combine all NMSCs into one study group [2].

Contrary to giant SCCs developing in anogenital regions, giant cSCCs arising in other locations are rarely reported [3]. In 2012, Wollina et al. [4] analyzed their collected data and performed research on all histologically confirmed non-melanoma skin cancers (NMSCs). In their database of about 2500 NMSCs, only 10 tumors (0.4%) fulfill the definition of giant cSCC [4]. Similarly to Wollina et al. [4], we observed seven giant cSCC cases in 6 years. In our opinion, internationally consistent cSCC registration practices are essential for progress against this cancer.

Phenotypic, genetic, and environmental factors contribute to the development of cSCC [1]. cSCC is more common in men than women [7]. The incidence rate is highest in fair-skinned individuals and major risk factors for cSCC development among this population are cumulative exposure to UV radiation in sunlight and advancing age [8]. In total, 80% of people diagnosed with cSCC are older than 60 [9]. Chronic inflammation and scarring are the principal causes of cSCC in individuals with highly pigmented skin [10]. Other significant causal factors include immunosuppression, human papillomavirus infection, ionizing radiation, inherited cancer syndromes, smoking, drugs, and exposure to carcinogens such as arsenic [1,9].

cSCC can become a giant tumor in case of delayed treatment due to not being noticed or being neglected by the patient [3]. The latter is commonly associated with advanced age, low socioeconomic status, poor hygiene, and fear of being diagnosed [11]. Otherwise, the slow-growing nature of most cases of cSCC can lead to the marginalization of tumors and prolong the time to treatment [11]. Furthermore, in 2019, Bajaj et al. [12] surveyed 173 skin cancer specialists in which they discovered that only 52% of participants routinely examined male genitalia, 21.28% female genitalia, and 34.7% the perianal area.

Some inflammatory disorders may clinically resemble cSCCs. There are case reports describing giant cSCCs complicating, e.g., psoriasis vulgaris, hidradenitis suppurativa (HS), lichen planus, folliculitis decalvans, or porokeratosis [13].

cSCC arising out of hidradenitis suppurativa is a rare, dreaded complication and diagnostic challenge due to the possible similar clinical presentations of both diseases [13]. The most common clinical presentation of HS-associated cSCC is a nonhealing ulcer. In 2017, Jourabchi et al. [14] came to the conclusion that HPV infection and smoking might be risk factors for developing cSCC in hidradenitis suppurativa. Despite the usually good differentiation of tumors, it is estimated that about half of the patients with HS-associated cSCC have metastatic dissemination of cancer leading to a poor prognosis [13]. On this account, an increased awareness is essential and repeated biopsies should be performed when cSCC development within an HS lesion is suspected [13].

In 2021, Sachdeva et al. [15] carried out a literature review of all cases of HS-associated cSCCs they found in the English literature. They analyzed the cases of 95 patients, with 122 cSCCs developed in pre-existing HS lesions in total. Overall, 77.9% (*n* = 74/95) of the patients were male. The mean age was 52.9 years. The SCCs were localized most commonly in the gluteal region (47.5%, *n* = 58/122), followed by the perianal area (18.9%, *n* = 23/122) and genitals (13.9%, *n* = 17/122). The mean time from HS onset to SCC diagnosis was 25.5 years. In 54% of patients (*n* = 34/63) metastases were reported, 43.1% (*n* = 28/65) had recurrence, and 58.7% (*n* = 44/75) died, 34.1% (*n* = 15/44) due to metastases and 13.6% (*n* = 6/44) because of sepsis [15].

Because of the rarity of HS-associated cSCC, no guidelines for treatment are well established. Considering the aggressive biological behavior of cSCCs developed in pre-existing HS lesions, high rates of metastases, and death of disease, timely excision with at least 20 mm margins may be the treatment of choice. Other therapeutic options include Mohs surgery and radiotherapy. Chemotherapy is not currently recommended as there is no proof of effectiveness [15].

The history of our patient is consistent with the above. Patient 1 was 59 years old and cSCC developed in a pre-existing Hurley stage III HS lesion 20 years after onset of the disease (Figure 1D). Nodal metastases were observed at the time of the diagnosis. His smoking history was also positive. As the percentage of deaths in this group of patients is high, this patient died despite the treatment. Although cSCCs arising out of HS lesions are rare, the presentation of the cSCCs in this group of patients may mimic HS. In patients suffering from HS, with nonhealing ulcers, around 20 years after the onset of hidradenitis suppurativa, this diagnosis should be taken into consideration in clinical practice.

In research performed by Wollina et al. [4], all patients with giant cSCCs were male. The mean age was 79.5 ± 11.4 years (mean ± standard deviation). The SCCs were localized on the scalp (eight), hand (one), and lower leg (one). The mean time to diagnosis was 25.2 ± 14.4 months. Neglect by patients was the most important cause of latency to treatment. In one patient, metastasis in-transit was reported. The mean size of skin lesions was 38.0 ± 76.0 cm^2^. Three cases were histologically defined as desmoplastic and one tricholemmal subtype was detected. Seven tumors were low-grade and three were intermediate [4].

In 2021, van Dam et al. [3] performed a systematic literature search on extra-anogenital cSCCs and analyzed 42 cases they identified. In total, 24 (57.1%) out of 42 patients were male. The mean age was 66.62 ± 21.33 years (mean ± standard deviation). The SCCs were localized on the face (13), scalp (13), lower limb (4), thoracic wall (3), upper limb (2), shoulder (2), back (1), hip (1), buttock (1), neck (1), and breast (1) [3].

cSCC has the highest estimated rate of mutations in comparison with other cancers. The most frequently mutated genes in cSCC are TP53, NOTCH, RAS, and CDKN2A [1].

The diagnostic process includes a clinical and dermatoscopic examination as well as a skin lesion biopsy [9]. cSCCs can arise on any cutaneous surface [6]. In individuals with a fair complexion, cSCCs occur most commonly in areas of chronic sun exposure, particularly the head and neck [9]. Giant cSCCs typically arise on sun-exposed skin as well [16]. In contrast, among dark-skinned people, cSCCs usually develop on sun-protected sites and areas of chronic inflammation and scarring, mostly affecting the legs and anogenital regions [10].

The clinical appearance of invasive cSCC depends on multiple factors such as lesion size, location, pigmentation, differentiation, and skin phototype. Well-differentiated cSCCs usually appear as verrucous and hyperkeratotic lesions. In contrast, poorly differentiated lesions are typically without hyperkeratosis and frequently have ulcerations [9]. There are case reports in the literature of giant cSCCs manifested as cutaneous horns [3].

cSCC can progress to locally advanced (lacSCC) and metastatic stages (mcSCC). LacSCCs are bigger, firm lesions and tend to spread to nearby tissues. McSCCs form in-transit, lymph node, and distant metastases [9]. The main sites of distant metastasis are lung, liver, bone, and brain [17].

Invasive cSCCs are mostly asymptomatic but accompanying symptoms like pain, pruritus, and local neurologic symptoms may be present [9,18].

The most important dermatoscopic features of invasive cSCC are white circles, white structureless areas, keratin, and blood spots [19]. Notwithstanding, the absence of white-colored structures, the presence of bleeding, and an increased quantity of small vessels within the lesion are potent predictors of poorly differentiated cSCC [20].

Skin biopsy with subsequent histological confirmation is essential to diagnose cSCC [6]. The selection of the biopsy technique depends on a chosen treatment option. Multiple histological variants of invasive cSCC exist, including those specified in the WHO classification of skin tumors [9].

Once the diagnosis of cSCC is established, the assessment of the risk of recurrence and metastasis is the most important step to choose the proper treatment approach. It involves history and physical examination, and in some cases imaging for local invasion and metastasis [9].

cSCC generally has a good prognosis with 5-year survival rates of greater than 90% and low estimated risks of local recurrence (3%), metastasis to lymph nodes (0.5–10%), distant metastasis (4–5%), and disease-specific death (1.5%) [3,9,21]. However, the outcome of cSCCs presenting features associated with aggressive behavior is worse and correlated with a higher risk of local recurrence and metastasis [22]. cSCCs in the metastatic stage have mortality rates above 70% [23]. Considering the above, efforts to drive progress in the early detection of cSCC are needed to improve patient outcomes.

cSCC with a diameter exceeding 20 mm is identified as a risk factor for recurrence, metastasis, and disease-specific death. In 2016, Thompson et al. [24] carried out a systematic review and meta-analysis of 36 studies (31 retrospective and 5 prospective cohort studies) including 17,248 patients and 23,421 cSCCs. The authors found a statistically significant association between cSCCs > 20 mm and risk for recurrence (RR, 3.22; 95% CI, 1.91–5.45), metastasis (RR, 6.15; 95% CI, 3.56–10.65), and disease-specific death (RR, 19.10; 95% CI, 5.80–62.95) [24]. 

It is important to highlight that a diameter of cSCC >20 mm was associated with the highest risk ratio for disease-specific death, followed by poor differentiation and location on the ear, whereas it is placed fourth and fifth in recurrence and metastasis risk assessment subcategories, respectively [24]. This implies that patients with giant cSCC compared to smaller tumors have worse outcomes.

In the literature review of van Dam et al. [3] of 42 cases of giant extra-anogenital cSCCs, in one patient nodal metastasis was reported, one patient had distant metastasis at diagnosis, and in four cases distant metastases occurred later.

The selection of a management strategy is influenced by many factors including cSCC-related complications such as the risk of recurrence, nodal and distant metastasis, and death. LacSCC and mcSCC are a priori considered as high-risk cSCCs. Remaining localized cSCCs should be categorized in one of the following groups: low-risk and high-risk cSCCs. Moreover, cancer staging is obtained before proceeding to treatment [9]. There are plenty of risk stratification and staging methods, e.g., AJCC TNM, UICC TNM, BWH T, NCCN, and EADO, but there are no universally accepted systems [6].

According to the mentioned data and stratification systems, all giant cSCCs are a priori considered high-risk because of their maximum clinical diameter determined by definition as ≥5 cm.

Imaging for regional nodal metastasis (US, CT), local invasion (MRI, CT), and distant metastasis (CT, PET-CT) is important for choosing a proper therapeutic approach. Giant cSCCs are more likely to be locally advanced or metastatic [9].

In general, surgical excision is the first-line therapy for most patients with invasive cSCC. However, in some cases, other modalities, such as nonsurgical destructive treatment (e.g., cryotherapy), radiotherapy, systemic immune checkpoint inhibitor therapy, and chemotherapy, are also utilized [25].

Considering the rarity of extra-anogenital giant cSCCs and the absence of prospective randomized trials, consistent recommendations for medical management do not exist. The proper approach to the management of giant cSCC should take into consideration multiple factors, such as tumor characteristics and patient-related variables, e.g., comorbidities and acceptance of the proposed therapy [3]. Likewise, for smaller cSCCs, surgery is the primary mode of treatment for giant tumors [3]. Standard excision with wide margins of 6–10 mm or micrographically controlled excision are the most recommended therapeutic options for high-risk cSCC. For confirmed nodal metastasis, lymph node dissection is needed [25]. The complete removal of the tumor with aesthetic and functional outcomes that are acceptable to the patient can be challenging in some patients with giant cSCCs, considering the size of the lesions and common locations such as the face and neck; consequently, in some cases, disabling surgery to obtain R0 margins is needed. Due to the size of giant cSCCs, reconstructive procedures such as graft closure are often needed to fix defects caused by surgery. Additional factors, e.g., advanced disease, and comorbid conditions may disqualify patients from surgery. Other therapeutic options such as radiotherapy and immunotherapy should be considered in those cases [25].

There is no proof that adjuvant therapies improve the survival rate in patients diagnosed with giant cSCC. However, adjuvant radiotherapy may be performed as a salvage therapy for patients with incompletely resected tumors not amenable to further surgery [3]. 

For patients with locally advanced or metastatic cSCC who are not candidates for curative surgery or radiotherapy, anti-PD-1 inhibitors are the treatment of choice. Cemiplimab was officially approved in 2018 by the FDA and in 2019 by the EMA for this indication [25]. Cetuximab, an EGFR inhibitor, combined with chemotherapy or radiotherapy may be considered as a second-line therapy for advanced cSCC [25].

Chemotherapy alone or in combination with EGFR inhibitors or radiotherapy can be a therapeutic option for patients with locally advanced or metastatic SCC not responding or intolerant to anti-PD1 agents. Platinum-based drugs are favored [25].

According to the analysis of giant cSCCs carried out by van Dam et al. [3] in 2021, radical surgical excision was the most common treatment approach—in 81% (*n* = 34/42) of cases, with 94% achieving R0 margins (*n* = 32/34)—regardless of the large size and the locations of the tumors mainly in the head and neck regions. Adjuvant radiotherapy was used only in two cases. Four patients were not candidates for curative surgery due to intracranial growth, high vascularization, distant metastasis, and a combination of advanced age, size, and location of the tumor; accordingly, they received supportive care. A patient with metastatic disease received chemotherapy and died a month later due to pulmonary metastases. Furthermore, photodynamic therapy was used in two cases. No patient received immunotherapy. Data about the risk factors for developing cSCC were not available [3]. 

In only 30 cases, follow-up was reported with a median time of 12 months. In the provided case outcomes, four patients (*n* = 4/30, 13%) died of disease 1, 3, 10, and 18 months after diagnosis was established, respectively. Two patients (*n* = 2/30, 7%) are alive with disease. Two cases (*n* = 2/30, 7%) of death of intercurrent illness were reported. In two cases, local recurrences were noted 1 and 3 months after surgery, respectively [3].

Our data also showed that in over 85% of cases, surgical excision was the treatment of choice. In our study group, neither of our patients underwent adjuvant radiotherapy. In contrast to the reported data, in our study group, three patients received immunotherapy. Moreover, we noted two cases of local recurrence similar to van Dam et al. We noticed one death of disease, four patients are alive without disease, and two are alive with disease and currently receive immunotherapy. The majority of recurrences or metastases are apparent within the first five years following the treatment of an initial cSCC; thus, close follow-up is required [3]. Patients with high-risk cSCC should have a clinical examination every 3–6 months for the first 2 years and every 6–12 months for years 3–5 and annually thereafter. In cases of advanced tumors, patients should have a clinical examination every 3 months for the first 5 years and every 6–12 months thereafter [25]. In patients with high-risk cancer, ultrasonography of lymph nodes is recommended every 3–6 months for 2 years depending on risk assessment and previous findings. For locally advanced and metastatic cSCC, ultrasonography is recommended every 3–6 months for 5 years and then every 6–12 months [25]. In advanced cases, imaging with CT, MRI, or PET-CT should be performed every 3–6 months for the first 3 years and then based on risk assessment [25].

As cumulative exposure to UV radiation in sunlight is the most common risk factor for cSCC development, sun protection is the main prevention method [3,8]. Giant cSCCs develop in cases where patients do not notice or neglect their symptoms [3]. This highlights the importance of cancer awareness campaigns, performing total body examinations, and improving access to health care.

We are aware of the limitations of our study. First of all, as we conducted a retrospective study, and we depended on patients’ medical records. To mitigate bias, we employed inclusion and exclusion criteria to ensure the homogeneity of our study population and minimize the influence of extraneous variables on our findings. Despite the retrospective nature of this study, all described cases were examined and treated surgically and/or with immunotherapy by authors. To ensure the accuracy and completeness of the data to reduce information bias, at least two team members analyzed the patients’ histories. However, the small number of presented cases may limit the generalization of our observations. 

## 5. Conclusions

Patients with giant cSCCs compared to smaller tumors have worse outcomes. The early detection and prevention of modifiable risk factors, such as UV radiation in sunlight, are essential. Total surgical excision is the treatment of choice. However, due to the size of tumors, their common location in the head area, patients’ advanced age, and the risk of disfigurement, treatment is difficult and requires a multidisciplinary approach. Accordingly, other therapies, including immunotherapy, may become increasingly important. In this study, we presented our observations on the use of cemiplimab and RP1 in three patients. As giant extra-anogenital cSCCs are rare, they have mostly been described as case reports or case series. Our data enrich the global dataset with additional observations regarding this patient group. At the same time, we would like to emphasize that there is a need for prospective studies involving a larger patient cohort. Multicenter research should be considered to gather the mentioned patient group. It may help to identify risk factors and help to compare different treatment modalities to achieve the best treatment outcomes. The risk factors observed in our study overlap with general risk factors for SCC. However, patients’ reported disease neglect may have also contributed to the delayed diagnosis and significant disease advancement. Therefore, future psychodermatological studies addressing the fear of diagnosis may contribute to identifying particularly vulnerable patient groups. 

## Figures and Tables

**Table 1:** Cases of extra-anogenital giant cutaneous squamous cell carcinomas in the years 2016–2022.

No.	Age (years)	Sex	Tumor Site	Maximum Clinical Diameter (mm)	Size of the Tumor (cm^2^)	Risk Factors	Time to Diagnosis (months)	Reason for a Prolonged Time to Diagnosis
1	59	M	Right buttock (Figure 1D)	103 mm	271.81 cm^2^	Hidradenitis suppurativa, smoking	18 months	Neglection; the similarity between cSCC and HS
2	68	M	Left supraclavicular area (Figure 2)	181 mm	562.94 cm^2^	Smoking	26 months	Neglection, fear of being diagnosed
3	84	M	Scalp (Figure 1C)	115 mm	390.19 cm^2^	UV radiation	12 months	Neglection
4	86	M	Scalp	86 mm	208.04 cm^2^	UV radiation, smoking	36 months	Neglection, fear of being diagnosed
5	89	M	Scalp (Figure 1A)	81 mm	160.32 cm^2^	UV radiation	43 months	Neglection, fear of being diagnosed
6	83	F	Left cheek (Figure 1B)	62 mm	114.92 cm^2^	UV radiation	59 months	Neglection, fear of being diagnosed
7	93	M	Left temple	84 mm	208.48 cm^2^	UV radiation	51 months	Neglection, fear of being diagnosed

M—male; F—female; HS—hidradenitis suppurativa; cSCC—cutaneous squamous cell carcinoma.

**Table 2:** Instances of giant cutaneous squamous cell carcinomas outside the anogenital region from 2016 to 2022.

No.	Histologic Subtype	Nodal Metastasis and/or in-Transit	Distant Metastasis	Treatment	Stage (TNM UICC/AJCC 8th Edition)	Outcome
1	Verrucous	Yes, nodal metastasis	No	Cemiplimab and RP1 injection	T3N1M0	DOD
2	Verrucous	Yes, nodal metastasis	No	Surgery + lymph node dissection + cemiplimab and RP1 injection	T3N1M0	AWD, during therapy
3	Verrucous	No	No	Surgery + 2xRT + cemiplimab and RP1 injection	T3N0M0	AWD, during therapy
4	Verrucous	No	No	Surgery (graft), R0	T3N0M0	NED
5	Verrucous	Yes, nodal metastasis	No	Surgery (graft), R0 + lymph node dissection	T3N1M0	NED
6	Verrucous	No	No	Surgery (primary closure), R0	T3N0M0	NED
7	Verrucous	No	No	Surgery (graft), R0	T3N0M0	NED

Abbreviations: NED—no evidence of disease; AWD—alive with disease; DID—death of intercurrent disease; DOD—death of disease; RT—radiotherapy; R0—microscopically margin-negative resection; RP1—genetically-modified HSV-1 oncolytic immunotherapeutic agent.

## Data Availability

The data generated in this study are available upon reasonable request from the corresponding author.

## References

[1] Que S.K.T., Zwald F.O., Schmults C.D. (2018). Cutaneous squamous cell carcinoma: Incidence, risk factors, diagnosis, and staging. J. Am. Acad. Dermatol..

[2] Lomas A., Leonardi-Bee J., Bath-Hextall F. (2012). A systematic review of worldwide incidence of nonmelanoma skin cancer. Br. J. Dermatol..

[3] Van Dam V., Trinh X.B., An B., Julien L. (2021). Extra-anogenital giant cutaneous squamous cell carcinomas require multidisciplinary management. Cancer Treat. Res. Commun..

[4] Wollina U., Bayyoud Y., Krönert C., Nowak A. (2012). Giant epithelial malignancies (Basal cell carcinoma, squamous cell carcinoma): A series of 20 tumors from a single center. J. Cutan. Aesthetic Surg..

[5] Nagarajan P., Asgari M.M., Green A.C., Guhan S.M., Arron S.T., Proby C.M., Rollison D.E., Harwood C.A., Toland A.E. (2019). Keratinocyte Carcinomas: Current Concepts and Future Research Priorities. Clin. Cancer Res..

[6] Kim J.Y.S., Kozlow J.H., Mittal B., Moyer J., Olenecki T., Rodgers P., Alam M., Armstrong A., Baum C., Work Group (2018). Guidelines of care for the management of cutaneous squamous cell carcinoma. J. Am. Acad. Dermatol..

[7] Xiang F., Lucas R., Hales S., Neale R. (2014). Incidence of nonmelanoma skin cancer in relation to ambient UV radiation in white populations, 1978–2012: Empirical relationships. JAMA Dermatol..

[8] Christenson L.J., Borrowman T.A., Vachon C.M., Tollefson M.M., Otley C.C., Weaver A.L., Roenigk R.K. (2005). Incidence of basal cell and squamous cell carcinomas in a population younger than 40 years. JAMA.

[9] Stratigos A.J., Garbe C., Dessinioti C., Lebbe C., Bataille V., Bastholt L., Dreno B., Fargnoli M.C., Forsea A.M., Frenard C. (2020). European interdisciplinary guideline on invasive squamous cell carcinoma of the skin: Part 1. epidemiology, diagnostics and prevention. Eur. J. Cancer.

[10] Higgins S., Nazemi A., Chow M., Wysong A. (2018). Review of Nonmelanoma Skin Cancer in African Americans, Hispanics, and Asians. Dermatol. Surg..

[11] Ricci F., Paradisi A., Fossati B., Mancini M., Curatolo P., Guerriero C., Capizzi R. (2015). Giant neglected squamous cell carcinoma of the skin. Dermatol. Ther..

[12] Bajaj S., Wolner Z.J., Dusza S.W., Braun R.P., Marghoob A.A., DeFazio J. (2019). Total Body Skin Examination Practices: A Survey Study Amongst Dermatologists at High-Risk Skin Cancer Clinics. Dermatol. Pract. Concept..

[13] Özkur E., Altunay İ.K., Celayir M.F., Çerman A.A., Uçak R. (2020). A Giant Squamous Cell Carcinoma Arising in a Patient with Hidradenitis Suppurativa. Adv. Skin Wound Care.

[14] Jourabchi N., Fischer A.H., Cimino-Mathews A., Waters K.M., Okoye G.A. (2017). Squamous cell carcinoma complicating a chronic lesion of hidradenitis suppurativa: A case report and review of the literature. Int. Wound J..

[15] Sachdeva M., Mufti A., Zaaroura H., Abduelmula A., Lansang R.P., Bagit A., Alhusayen R. (2021). Squamous cell carcinoma arising within hidradenitis suppurativa: A literature review. Int. J. Dermatol..

[16] Alam M., Ratner D. (2001). Cutaneous squamous-cell carcinoma. N. Engl. J. Med..

[17] Sparsa A., Doffoel-Hantz V., Durox H., Gaston J., Delage-Core M., Bédane C., Labrousse F., Sannajust J.-P., Bonnetblanc J.-M. (2012). Tumeur maligne historique: 27 cas [Historic malignant tumour: 27 observations]. Ann. Dermatol. Venereol..

[18] Reule R.B., Golda N.J., Wheeland R.G. (2009). Treatment of cutaneous squamous cell carcinoma with perineural invasion using Mohs micrographic surgery: Report of two cases and review of the literature. Dermatol. Surg..

[19] Rosendahl C., Cameron A., Argenziano G., Zalaudek I., Tschandl P., Kittler H. (2012). Dermoscopy of squamous cell carcinoma and keratoacanthoma. Arch. Dermatol..

[20] Lallas A., Pyne J., Kyrgidis A., Andreani S., Argenziano G., Cavaller A., Giacomel J., Longo C., Malvestiti A., Moscarella E. (2015). The clinical and dermoscopic features of invasive cutaneous squamous cell carcinoma depend on the histopathological grade of differentiation. Br. J. Dermatol..

[21] Karia P.S., Morgan F.C., Califano J.A., Schmults C.D. (2018). Comparison of Tumor Classifications for Cutaneous Squamous Cell Carcinoma of the Head and Neck in the 7th vs 8th Edition of the AJCC Cancer Staging Manual. JAMA Dermatol..

[22] Kwon S., Dong Z.M., Wu P.C. (2011). Sentinel lymph node biopsy for high-risk cutaneous squamous cell carcinoma: Clinical experience and review of literature. World J. Surg. Oncol..

[23] Burton K.A., Ashack K.A., Khachemoune A. (2016). Cutaneous Squamous Cell Carcinoma: A Review of High-Risk and Metastatic Disease. Am. J. Clin. Dermatol..

[24] Thompson A.K., Kelley B.F., Prokop L.J., Murad M.H., Baum C.L. (2016). Risk Factors for Cutaneous Squamous Cell Carcinoma Recurrence, Metastasis, and Disease-Specific Death: A Systematic Review and Meta-analysis. JAMA Dermatol..

[25] Stratigos A.J., Garbe C., Dessinioti C., Lebbe C., Bataille V., Bastholt L., Dreno B., Fargnoli M.C., Forsea A.M., Frenard C. (2020). European interdisciplinary guideline on invasive squamous cell carcinoma of the skin: Part 2. Treatment. Eur. J. Cancer.

